# Sun moths in the genus *Epicroesa* (Lepidoptera, Heliodinidae) associated with *Ceodes umbellifera*, with description of a new species from Taiwan

**DOI:** 10.3897/zookeys.1289.194186

**Published:** 2026-08-11

**Authors:** Yu-Feng Hsu

**Affiliations:** 1 Department of Life Science, National Taiwan Normal University, Taipei, 116, Taiwan Department of Life Science, National Taiwan Normal University Taipei Taiwan

**Keywords:** Australia, disjunct distribution, *Epicroesa bellatula* sp. nov., heliodinid, host plants, immature biology

## Abstract

The association of two heliodinid moths with *Ceodes umbellifera* is documented for the first time: *Epicroesa thiasarcha* Meyrick, 1907 from Australia and *E. bellatula***sp. nov**. from Taiwan. The two share similar wing patterns and genitalia, but they can be separated by diagnostic features of adults and immatures. The *p*-distance of COI barcodes was over 9% between samples of the two taxa, lending support to their recognition as distinct species. The considerable geographical separation between these morphologically similar yet genetically diverged species suggests that either both may have broader distributions than currently recognized, or additional undescribed species may occur in the large gap between Australia and Taiwan.

## Introduction

Although moths in the family Heliodinidae, the sun moths, are distributed primarily in the New World, the genus *Epicroesa* is known only from the Old World (Hsu and Powell 2005). Six described species are currently recognized: *E. ambrosia* Meyrick, 1907, *E. calliteucha* Meyrick, 1912, *E. chromatorhoea* Diakonoff & Arita, 1979, *E. metallifera*, Meyrick, 1907, *E. thiasarcha*, Meyrick, 1907, and *E. lylae* Heppner, 2021. Of them, *E. chromatorhoea* is endemic to Japan (Yasuda 2013), *E. lylae* is known from Tahiti (Heppner 2021), and the remaining four are documented from Australia and New Guinea (Hsu and Powell 2005). Hsu and Powell (2005) listed diagnostic characters of the genitalia for the genus.

Yen et al. (2022) reported *E. thiasarcha* from Taiwan. Because this species was previously considered an Australian endemic, the population in Taiwan would seem to represent a rare case of amphitropical distribution for Heliodinidae. However, after a closer examination of the material from Taiwan and Australia, considerable differences are evident in wing patterns, genitalia, and DNA barcodes between the samples from the two regions. Consequently, samples from Taiwan are recognized as a new species, *Epicroesa bellatula* sp. nov., which is described here.

Heliodinid moths are specialists as larvae, with approximately 75% of those for which larval hostplants are known feeding on Nyctaginaceae (Hsu and Powell 2005). The larval hostplant of *E. thiasarcha* has been unrecorded until now. Both *E. thiasarcha* and *E. bellatula* sp. nov. were found to use the same species of Nyctaginaceae, formerly referred to as *Pisonia umbellifera* J.R.Forst & G.Forst (e.g. Stemmerik 1964; Quisumbing 1978; Lavaud et al. 1996; Tang et al. 1996; Yang and Lu 1996; Pramanick et al. 2015). However, Rossetto et al. (2019) discovered that the genus *Pisonia* sensu lato was not monophyletic. Rossetto and Caraballo-Ortiz (2020) subsequently reclassified *Pisonia*, dividing it to three genera, *Pisonia*, *Rockia*, and *Ceodes*, with *P. umbellifera* assigned to *Ceodes*. The larval hostplant association of *Ceodes umbellifera* for heliodinid moths is reported here for the first time, and taxonomic accounts are given for the two species.

## Materials and methods

Procedures for genitalic dissection and terminology for wing pattern and venation follow Hsu and Powell (2005). Terminology for genitalia follows Klots (1970). Vouchers are deposited in the Department of Life Science, National Taiwan Normal University, Taipei (**NTNU**), National Museum of Natural Science, Taichung (**NMNS**), Natural History Museum of London, U.K. (**NHMUK**), and the Australian National Insect Collection, Canberra, Australia (**ANIC**). Immatures were reared in plastic containers (15 × 8 × 4.5 cm). Rearing codes follow the system of Powell and De Benedictis (1995). For example, in “HSU 03E9,” “HSU” refers to the the name of the rearing database; “03” refers to 2003; “E” refers to the fifth month of the year (E = May), and “9” refers to the ninth collection in May 2003. A single adult of *Epicroesa* from Taiwan and another from Australia were used for DNA extraction. Genomic DNA was extracted from the whole body of each *Epicroesa* individual. After each specimen was ground separately in an Eppendorf tube using a plastic pestle, the homogenized tissue was digested in cell lysis buffer at 60 °C for 3 h. DNA was then extracted using a NautiaZ Tissue DNA Mini Kit (Nautia Gene, Taipei, Taiwan) following the manufacturer’s protocol. A 665-bp fragment of the mitochondrial cytochrome c oxidase subunit I (COI) gene was amplified using the universal primer pair LCO1490 (5'-GGTCAACAAATCATAAAGATATTGG-3') and HCO2198 (5'-TAAACTTCAGGGTGACCAAAAAATCA-3') (Folmer et al. 1994). Polymerase chain reactions (PCRs) were performed using Ready-To-Go™ RT-PCR Beads (Cytiva, USA) in a total volume of 25 μl, containing 1 μl each of forward and reverse primers (10 μM), 2 μl of genomic DNA, and 21 μl of ddH2O. PCR conditions consisted of an initial denaturation at 94 °C for 2 min, followed by 35 cycles of denaturation at 94 °C for 20 s, annealing at 50 °C for 40 s, and extension at 72 °C for 1 min, with a final extension at 72 °C for 10 min. Amplified products were purified using a NautiaZ Gel/PCR DNA Purification Mini Kit (Nautia Gene, Taipei, Taiwan). Sequencing was carried out on an ABI 3730XL DNA Analyzer at the DNA Sequencing Core Facility, Institute of Biomedical Sciences, Academia Sinica, Taipei, Taiwan. Sequences were visually checked and edited in BIOEDIT v. 7.0 (Hall 1999). Sequences were aligned using MUSCLE, and genetic differentiation was estimated as pairwise distances among the ten individuals under the Kimura 2-parameter model in MEGA 12 (Tamura et al. 2021). Both newly generated sequences were deposited in GenBank with detailed sample information.

## Results

The heliodinid moths associated with *Ceodes umbellifera* in Australia and Taiwan share similar wing patterns with orange tips of forewing apices ornamented with silver markings on the forewing upperside and lanceolate scales in coremata. Nonetheless, considerable differences are present in the wing pattern and genitalia. Diagnostic features were also recognized in markings on the head capsule of nearly mature larvae and in the shape of the pupa. The *p*-distance of the COI barcode was 9.75% (GenBank under the accession numbers PZ166696 and PZ166697), well over the often cited 3% COI genetic divergence Hebert et al. (2003) proposed for species delimitation criteria for Lepidoptera. Consequently, the morphological and molecular evidence both support the hypothesis that the two represent distinct species.

### Systematic account

#### Epicroesa

Taxon classificationAnimaliaLepidopteraHeliodinidae

Meyrick, 1907

D083E2CC-B36F-5682-94AD-43840923F9C1

Epicroesa Meyrick, 1907: 95. Type species: Epicroesa ambrosia Meyrick, 1907 by monotypy.

##### Diagnosis.

No sexual dimorphism in wing pattern. Forewing R4 and R5 separate; CuA2 present. Forewing ground colour metallic dark brown, tinged with deep purple, and variously mottled with metallic green, pink, silver, or orange, sometimes concentrated to form bands or markings, often metallic orange with distal silver spots. Frenulum with one bristle in male, two bristles in female and with one longer. Coremata containing slender scales. Cornuti consisting of numerous spines, larger cephalad, finer caudad. Signa forming prominent sclerotised patch armed with minute spines.

##### Distribution.

Australian, Palaearctic, Oriental, Afrotropical (Seychelles Islands), and Southern Pacific; seven described species (including one described in the present study).

#### Epicroesa thiasarcha

Taxon classificationAnimaliaLepidopteraHeliodinidae

Meyrick, 1907

492C7DFA-27CE-5DB3-9C96-1089EF5387AA

Figs 1, 3, 5, 7, 9, 11, 13, 14

Epicroesa thiasarcha Meyrick, 1907: 95. Type locality: Cairns, Queensland, [Australia]; Common 1990: 212.

##### Type material.

***Lectotype***. Hereby designated. [AUSTRALIA] • ♂; Queensland; Cairns; FPD; “9. 05 [September, 1905]” (examined); NHM. ***Paralectotype***. [AUSTRALIA] • 1♀; same locality as lectotype; “10. 05 [October, 1905]”; Meyrick collection; NHM (examined).

**Figures 1–6.**
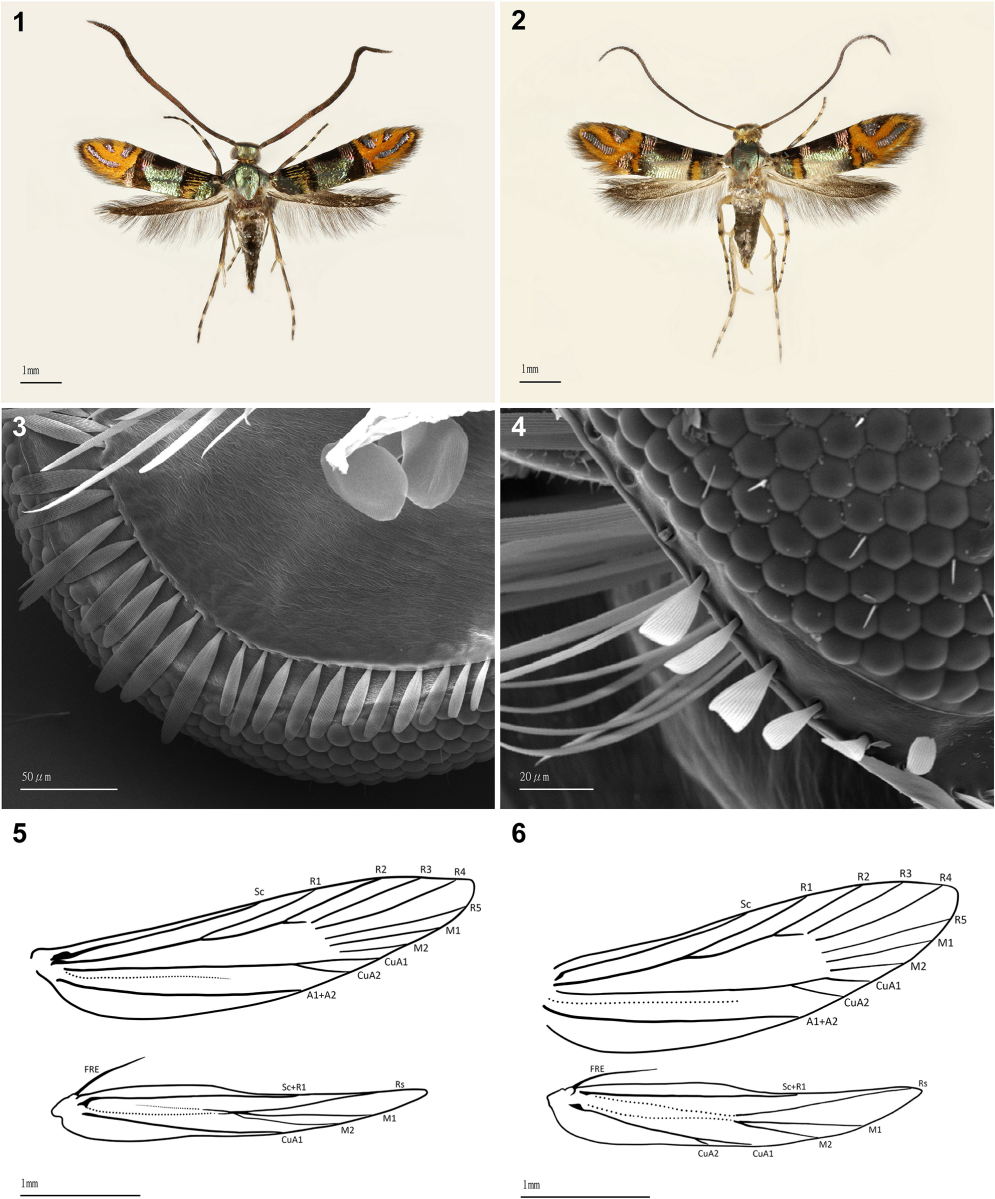
Adults of *Epicroesa* moths. **1**. *E. thiasarcha* male, Australia; **2**. *E. bellatula* sp. nov., female, holotype; **3**. Scaling behind eye of *E. thiasarcha*; **4**. Scaling behind eye of *E. bellatula* sp. nov.; **5**. Venation of *E. thiasarcha*; **6**. Venation of *E. bellatula*.

**Figures 7–12.**
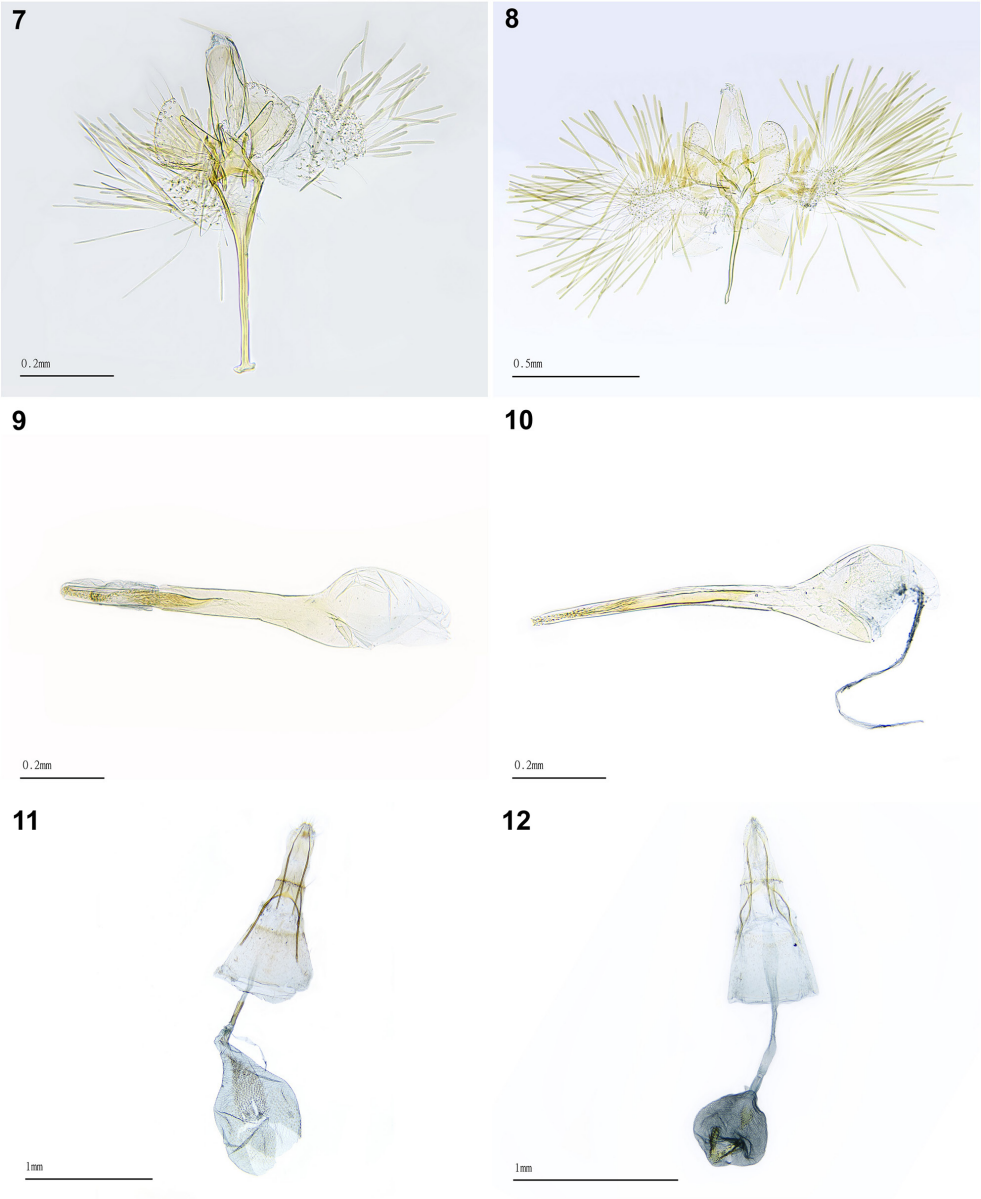
Genitalia of *Epicroesa* moths. **7**. Male genitalia of *E. thiasarcha*, with phallus removed; **8**. Male genitalia of *E. bellatula* sp. nov. with phallus removed; **9**. Phallus of *E. thiasarcha*; **10**. Phallus of *E. bellatula* sp. nov.; **11**. Female genitalia of *E. thiasarcha*; **12**. Female genitalia of *E. bellatula* sp. nov.

**Figures 13–18.**
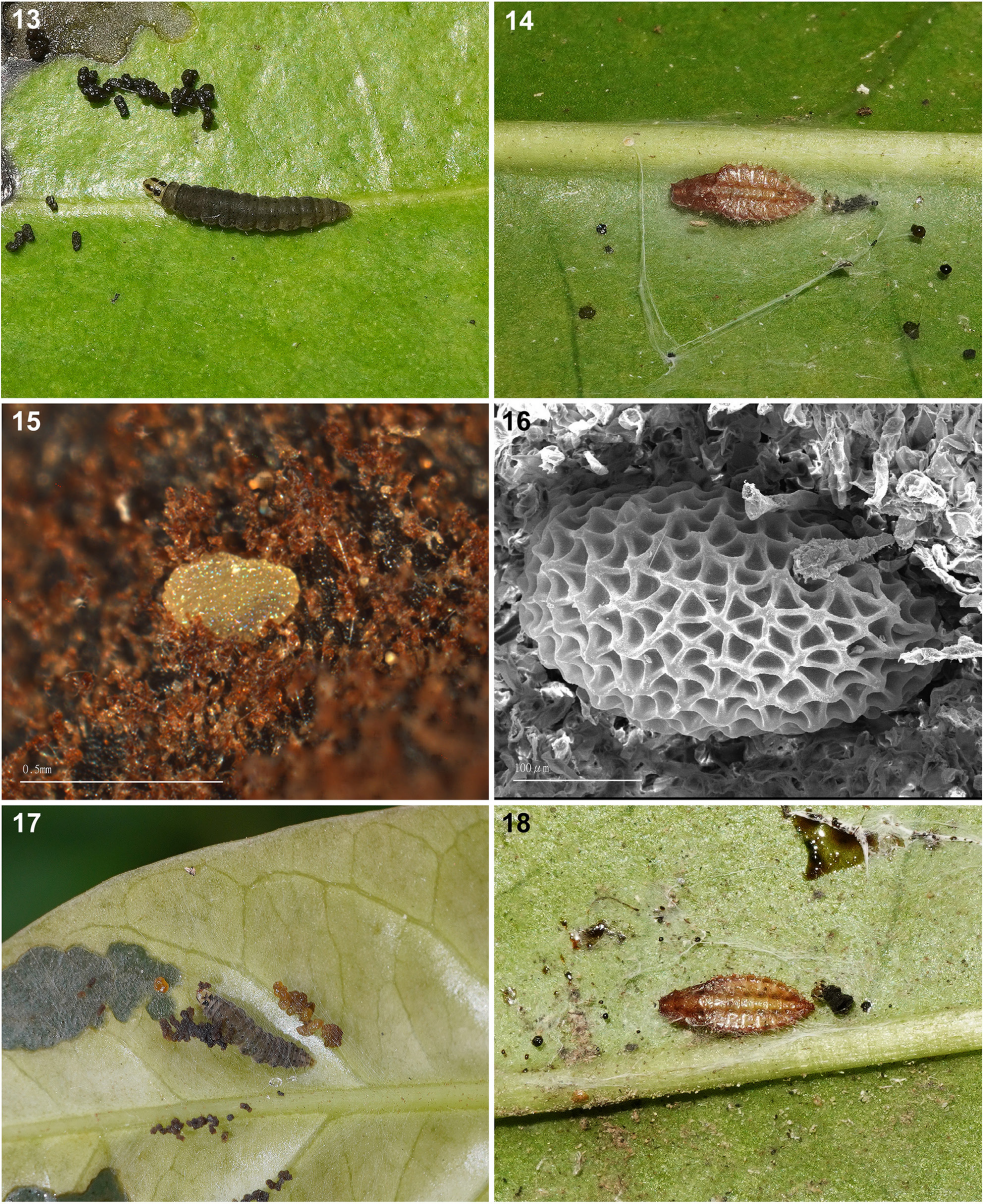
Immatures of *Epicroesa* moths. **13**. Mature larva of *E. thiasarcha*; **14**. Pupa of *E. bellatula* sp. nov.; **15**. Egg of *E. bellatula* sp. nov. **16**. SEM photograph of egg of *E. bellatula* sp. nov. **17**. Mature larva of *E. bellatula* sp. nov. **18**. Pupa of *E. bellatula* sp. nov.

##### Additional material.

[AUSTRALIA] • 1♂; Queensland; Cairns; FPD.; “10 [October, no year given]”; Meyrick collection; NHM • 1 ex [abdomen missing]; Queensland, Kuranda; 20 August 1904; Dodd [coll.]; Walsingham collection; NHM • 1♀; same data except date; 22 August 1904; Dodd [coll.]; Walsingham collection; NHM • 1♂, 1♀; 17 October 1904; Dodd [coll.]; Walsingham collection; NHM •1♂; Queensland; Bellender Ker; 27 March 2017; reared from *Ceodes umbellifera*; emgd. 6 April 2017; HSUM 17C28, Y. F. Hsu, M. F. Braby coll.; ANIC •. 2♀; same data except date; 23 October 2023; reared from *C. umbellifera*; emgd. 13–17 February 2023; HSUM 23B7, Y. F. Hsu coll.; ANIC • 1♀; Queensland; 4.5 km W. of Nerada; 31 January 2023; reared from *C. umbellifera*; emgd. 14 February 2023; HSU 23A29; Y. F. Hsu, Y. C. Lin, M. F. Braby coll.; ANIC • 4♂, 1♀; 4 February 2023; reared from *C. umbellifera*; emgd. 13–17 February 2023; HSUM 23B7; Y. F. Hsu, Y. C. Lin, M. F. Braby coll.; ♀ genitalia preparation YYL 0045; ANIC • 1♂, 2♀; 27 February 2024; reared from *C. umbellifera*; emgd. 11–17 March 2024; HSU 23B14; Y. F. Hsu coll.; ANIC • 5♂, 4♀; 23 October 2024; reared from *C. umbellifera*; emgd. 5–8 November 2024; HSU 24B24.1; 1♂ genitalia preparation YYL 0048; Y. F. Hsu coll.; ANIC.

##### Redescription.

***Head***: smooth scaled, grey, tinged with metallic blue-green. Scales posteriad of compound eye dark brown, elongate, lanceolate. Labial palpus drooping, straight, dark brown. Haustellum unscaled. Antenna slightly longer than forewing length, filiform but slightly flattened, with dark brown scales. ***Thorax***: dorsum silvery green tinged with blue. Tegulae elongate, prominent, lobate, with length over half of mesothoracic tergum. Legs creamy yellow, shiny proximally, silvery banded, distally with dark brown; tibial spurs 0-2-4, with inner spurs longer than outer; bristle-like scales on metatibiae. Forewing length 3.05–3.94 mm (mean = 3.55 ± 0.17 mm, *n* = 30); forewing lanceolate, with obtuse apex; hindwing narrower than forewing, with vestigial CuA2. Ground colour on basal 2/3 of forewing upper side dark brown, overlaid with a basal and a medial, metallic-green patch tinged with blue. Medial patch surrounded by three dark-brown bands, one costal, one proximal, and one distal, with proximal band ornamented with orange streaks; distal 1/3 orange. Four costal, one apical, and one dorsal, metallic-silver, purple-tinged spots; apical spot elongate, curved, remaining spots dentate or bar-shaped; size formula: C2 > D1 > C1 > C3 > C4. Fringe brown. Upper side of hindwing distally brown, proximally white. ***Abdomen***: dark brown, dorsally shiny, slivery banded, with dark brown ventrally, creamy yellow caudad. Male genitalia (Figs 7, 9) with tegumen protruded posteriad, extending beyond socii. Vinculum fused with tegumen, inconspicuous. Saccus elongate, tapering cephalad, with distal knob, much longer than tegumen. Socius digitate, setose. Valva simple, setose, triangular, axe-shaped. Phallus elongate, enlarged basad, tapering posteriad. Cornuti consisting of numerous spicules, finer caudad. Coremata forming enormous pouches containing a bundle of lanceolate scales. Female genitalia (Fig. 11) with medial, ventral band of apophyses anteriores narrow, triangular; apophyses posteriors approximately equal in length to apophyses anteriores. Ductus bursae elongate, slightly sclerotised. Ductus seminalis originating near base of corpus bursae. Corpus bursae ovoid, posteriorly narrowed, membranous and with wrinkled wall. Signa forming a prominent elongate, bifurcate, sclerotised band armed with prominent spines; one as smaller sclerite with short cones.

##### Early stages.

(Figs 13, 14) Egg not observed. Last instar 6.07 ± 0.39 mm (*n* = 16) in length (Fig. 13), elongate, eruciform, tapering toward blunt caudal end. Head rounded, with deeply V-shaped medial notch on vertex; head capsule ivory-coloured, with a pair of prominent dark-brown, straight bands on head capsule. Transparent setae present throughout body surface. Ground colour of body uniformly dark brown. Pupa 4.92 ± 0.09 mm long, 1.90 ± 0.09 mm (*n* = 13) wide at A3 (Fig. 14), flattened, posteriorly attenuate, with caudal end pointed; anteriorly tapering, with cephalic end blunt, bearing two pairs of small, bump-like processes. Abdomen with flat dorsal surface with prominent lateral ridge, with three lateral setae present at each segment from A2 through A7; one prespiracular and two postspiracular setae.

##### Biology.

The larval host plant is *Ceodes umbellifera*. The larva constructs a loose silk shelter on the underside of the leaf. Larvae skeletonise leaves, devouring mesophyll tissue, with the frass deposited nearby. Pupation occurs on the leaf underside in a loosely constructed cocoon. This species is apparently multivoltine because both adults and immatures can be found at different times of the year.

##### Distribution.

Australia (Queensland).

#### Epicroesa bellatula sp. nov.

Taxon classificationAnimaliaLepidopteraHeliodinidae

A56CA7B5-1C1B-59E2-BBEB-43953A1F9D5B

https://zoobank.org/A5ECC5AB-385A-495E-A992-F70798C291A1

Figs 2, 4, 5, 8, 10, 12, 15–18

Epicroesa thiasarca [sic]: Yen (not Meyrick 1907) 2022: 140.

##### Type material.

***Holotype***. TAIWAN • ♀; TAIDONG Co.; Lanyu, Sidaogou; 2 May 2003; reared from *Ceodes umbellifera*; emgd. 19 May 2003; HSU 03E9; Y. F. Hsu coll.; NHM. ***Paratypes***. TAIWAN • 12♂, 1♀; same locality, date as holotype; emgd. 13–21 May 2003; Y. F. Hsu coll.; NHM, NTNU, NMNS • 1♀; 21 March 2020; reared from *C. umbellifera*; emgd. 10 April 2020; HSU 20C40, Y. F. Hsu coll.; NTNU • 1♂, same data except date; 19 March 2023; reared from *C. umbellifera*; emgd. 5 April 2023; HSU 23C26; Y. F. Hsu coll.; genitalia preparation YYL0037; NTNU • 6♂, 4♀; TAIDONG Co.; Lanyu, Longman Bridge; 2 May 2003; reared from *C. umbellifera*; emgd. 12–15 May 2003; HSU 03E7; Y. F. Hsu coll.; NTNU, NMNS • 3♂, 5♀; same data except date; 13–14 June 2003; reared from *C. umbellifera*; emgd. 24 June–2 July 2003; HSU 03F9; Y. F. Hsu coll.; NTNU • 1♂ 1♀; TAIDONG Co.; Lanyu, Langdao; 14 June 2003; reared from *C. umbellifera*; emgd. 28 June–3 July 2003; HSU 03E7; Y. F. Hsu coll.; NTNU • 7♂, 4♀; same data except date; 22 March 2020; reared from *C. umbellifera*; emgd. 2–12 April 2003; HSU 20C49; Y. F. Hsu coll.; NTNU, NHM • 3♂; 2 May 2022; same data except date; reared from *C. umbellifera*; emgd. 13–16 May 2022; HSU 22E46; Y. F. Hsu coll.; NTNU; 4♂, 5♀; same data except date; 20 March 2023; reared from *C. umbellifera*; emgd. 5 April 2023; HSU 23C29; Y. F. Hsu coll.; NTNU; 3♀; JIAYI Co.; Alishan, Shanmei, Danaigu, 500 m; 4–6 May 2007; reared from *C. umbellifera*; emgd. 17 May 2007; HSU 07E12; Y. F. Hsu coll.; NTNU •1♂; 1♀; same data except date; 12 November 2016, reared from *C. umbellifera*, emgd. 26 November 2016; HSU 16L6; Y. F. Hsu coll.; NTNU • 1♀; KAOHSIUNG CITY; Jiaxian Dist., Butingshan; ca 500 m; 25 June 2020; reared from *C. umbellifera*; emgd. 12 July 2020; HSU 20F25; Y. F. Hsu coll.; NTNU • 1♀; same data except date; 29 December 2020; reared from *C. umbellifera*, emgd. 21 January 2021; HSU 20F25, Y. F. Hsu coll.; NTNU • 1♀, same data except date; 29 December 2020; reared from *C. umbellifera*; emgd. 21 January 2021; HSU 20M23; Y. F. Hsu coll.; genitalia preparation YYL0031; NTNU • 1♂; same data except date; 20 May 2023; reared from *C. umbellifera*; emgd. 1 July 2023; HSU 23E73; Y. F. Hsu coll.; NTNU.

##### Description.

***Head***: smooth scaled, grey, tinged with metallic blue. Scaling posteriad of compound eye creamy yellow, spatulate. Labial palpus drooping, straight, creamy yellow, dark brown at distal end. Haustellum unscaled. Antenna slightly longer than length of forewing, filiform, slightly flattened, with dark-brown scales. ***Thorax***: silvery, dorsally tinged with blue, ventrally creamy yellow. Tegulae elongate, prominent, lobate, with length over half of mesothoracic tergum. Legs creamy yellow, proximally shiny, silvery, distally banded with dark brown; tibial spurs 0-2-4, with inner spurs longer than outer; bristlelike scales on metatibiae. Forewing length 2.76–3.69 mm (mean = 3.39 ± 0.23 mm, *n* = 30); forewing lanceolate, with apex obtuse; hindwing narrower than forewing, with CuA2 present. Ground colour on basal 3/5 of forewing upper side dark brown, overlaid with a basal and a medial metallic-yellow, green-tinged patch. Medial patch surrounded by three dark-brown bands, one costal, one proximal, and one distal, with proximal band ornamented with orange marking; distal 2/5 orange. Three costal, one apical and one dorsal metallic-silvery, purple-tinged spots; apical spot elongate, straight, remaining spots dentate or bar-shaped; size formula: C2 > C3 > D1 > C1. Fringe brown. Upper side of hindwing distally brown, proximally white. ***Abdomen***: dark brown, shiny dorsally, creamy yellow with caudal end dark brown ventrally. Male genitalia (Figs 8, 10) with tegumen protruded posteriad, extending beyond socii. Vinculum fused with tegumen, inconspicuous. Saccus elongate, tapering cephalad, much longer than tegumen. Socii digitate, setose. Valva simple, setose, forming elongate, ovate lobe. Phallus elongate, enlarged basad, tapering posteriad. Cornuti consisting of numerous spicules, finer caudad. Coremata forming enormous pouches containing a bundle of elongate, lanceolate scales. Female genitalia (Fig. 12) with medial, narrow, ventral band of apophyses anteriores, posteriorly with needle-like protrusion; apophyses posteriors approximately equal to apophyses anteriores in length. Ductus bursae elongate, membranous. Ductus seminalis originating near base of corpus bursae. Corpus bursae bulbous. Signa double, larger one V-shaped, armed with prominent spines; smaller one with short cones.

##### Early stages.

(Figs 15–18) Egg 0.35 ± 0.03 mm long, 0.20 ± 0.01 mm in diameter (*n* = 6) (Figs 15, 16), ovoid, with fine sculpturing on surface of thin chorion. Length of mature larva 5.94 ± 0.39 mm (*n* = 22). Larva (Fig. 17) stout, tapering toward both ends, with caudal end blunt. Head rounded, with deep, medial, V-shaped notch on vertex. Head capsule ivory-coloured, with prominent dark-brown markings along ecdysial sutures and thick, straight bands laterally. Dark-brown setae present throughout body surface. Ground colour semitransparent, with extensive dark-brown markings tinged with purple dorsally throughout body. Pupa 4.63 ± 0.36 mm long, 1.68 ± 0.17 wide at A3 (*n* = 17), flattened, posteriorly attenuate, with caudal end pointed; anteriorly tapering, with cephalic end blunt, bearing two pairs of small, bump-like processes. Abdomen with flat dorsal surface with prominent lateral ridge, with three setae at each segment A2 through A7, one prespiracular and two postspiracular.

##### Etymology.

A diminutive form of an adjective of Latin origin, *bellatulus* = beautiful.

##### Biology.

The host plant is *Ceodes umbellifera*. The egg is laid on the leaf and flower buds, young twigs, or flower buds. The larva constructs a loose silk shelter and bores into flower buds or skeletonises the underside of leaves for feeding. The pupa is usually attached to the underside of the leaf. This species is apparently multivoltine because adults and immatures can be found throughout the year.

##### Distribution.

Specimens are known only from southern Taiwan.

##### Diagnosis.

*Epicroesa bellatula* sp. nov. is similar to *E. thiasarcha* in wing shape and pattern, but can be distinguished from the latter by the following characters: 1) the proximal dark-brown band on forewing upperside is overlaid by a few narrow, parallel, orange streaks in *E. thiasarcha* (Fig. 1), but an orange patch in *E. bellatula* sp. nov. (Fig. 2); 2) four metallic markings are present in the distal orange portion of the upper side of the forewing in *E. thiasarcha* (Fig. 1), in contrast to three in *E. bellatula* sp. nov. (Fig. 2); 3) metallic scaling of basal half of forewing and head is green, tinged with blue in *E. thiasarcha* (Fig. 1), but yellow, tinged with green in *E. bellatula* sp. nov. (Fig. 2); 4) hindwing vein CuA2 is vestigial in *E. thiasarcha* (Fig. 5), but present in *E. bellatula* (Fig. 6); 5) the valva is triangular in *E. thiasarcha* (Fig. 7), but elongate, lobate in *E. bellatula* sp. nov. (Fig. 8); 6) the signum forms an elongate, sclerotised band in *E. thiasarcha* (Fig. 11), but in the form of two V-shaped sclerites in *E. bellatula* sp. nov. (Fig. 12); 7) the head capsule of last instar larva bears a pair of dark-brown bands in *E. thiasarcha* (Fig. 13) compared to a Y-shaped, dark-brown mark in *E. bellatula* (Fig. 17); 8) the pupa of *E. thiasarcha* (Fig. 14) is wider than that of *E. bellatula* sp. nov. (Fig. 18), with average ratio of body length/width of A3 approximately 2.58× versus 2.75 (*n* = 30 for each species).

## Discussion

The two *Epicroesa* species associated with *Ceodes umbellifera* reported here appear to be more closely related to each other than their congeners, as they share similar wing patterns and genitalia. Their known distribution, Australia and Taiwan, however, are separated by more than 6000 km. A plausible explanation for such a vast gap in the distribution of closely related species may be that both are actually more widely distributed than currently recognised. An alternative explanation is that there may be additional *Ceodes umbellifera*-associated, undiscovered taxa in the vast area between Australia and Taiwan as the host plant, *C. umbellifera*, is widespread Southeast Asia and the South Pacific (Pramanick et al. 2015; Rossetto and Caraballo-Ortiz 2020).

Walsingham (1909) regarded the absence of hindwing vein CuA2 as a diagnostic character for the genera *Pseudastasia* and *Embola*. Hsu and Powell (2005) argued that the presence of the vein is a plesiomorphic character, as it occurs in the majority of basal lineages of heliodinids as well as in the selected outgroups in their study. They demonstrated the loss of CuA2 is homoplastic in Heliodinidae and assigned this character state to *Epicroesa* based on observation of the venation of *E. metallifera* Meyrick, 1907. In the present study, it appears that the presence/absence of hindwing CuA2 is not a reliable diagnostic character for the genus *Epicroesa*.

## Supplementary Material

XML Treatment for Epicroesa

XML Treatment for Epicroesa thiasarcha

XML Treatment for Epicroesa bellatula

## Additional information

### Artificial Intelligence (AI) use

The author accepts full responsibility for the content of the manuscript, including the disclosure of any use of AI.

No AI tools were used in the preparation of this manuscript.

### Author ORCIDs

Y.-F. Hsu https://orcid.org/0000-0003-2091-7775

### Data availability

All of the data that support the findings of this study are available in the main text.
